# A new species of genus *Hoplocryptus* Thomson (Hymenoptera, Ichneumonidae, Cryptinae) and a key to species from Oriental and Eastern Palaearctic regions

**DOI:** 10.3897/zookeys.865.35094

**Published:** 2019-07-22

**Authors:** Xi-Nan Wang, Mao-Ling Sheng, Martin Schwarz

**Affiliations:** 1 Research Institute of Forest Protection, Shandong Academy of Forestry, Jinan, Shandong 250014, China Research Institute of Forest Protection, Shandong Academy of Forestry Jinan China; 2 General Station of Forest and Grassland Pest Management, National Forestry and Grassland Administration, Shenyang, Liaoning, 110034, China National Forestry and Grassland Administration Shenyang China; 3 Eben 21, A-4202 Kirchschlag, Austria Unaffilaited Kirchschlag Austria

**Keywords:** Agrothereutina, Cryptini, Eastern Palaearctic region, key, Oriental region, taxonomy

## Abstract

A new species of Cryptinae, *Hoplocryptusqingdaoensis* Sheng, Wang & Schwarz, **sp. nov.** collected from Qingdao, Shandong Province, in the north border of oriental part of China, is described and illustrated. A key to species known from the Oriental and Eastern Palaearctic regions is provided.

## Introduction

*Hoplocryptus* Thomson, 1873 belongs to the tribe Cryptini of the subfamily Cryptinae (Hymenoptera: Ichneumonidae), and comprises 32 species (Yu et al. 2016), of which three are known from the Oriental Region (Chao 1976, Uchida 1931, 1940, 1956), 14 are from the Eastern Palaearctic Region (Momoi 1963, 1968, 1973, Schwarz 2007, Uchida 1936, 1952, 1956, Yu et al. 2016) (seven of them are found across the Palaearctic), 14 from the Western Palaearctic Region (Schwarz 2007, Yu et al. 2016) and nine from the Nearctic Region (Townes and Townes 1962, Yu et al. 2016). Schwarz (2007) reviewed West Palaearctic species of this genus with a key to these species. Up to now, four species of *Hoplocryptus* Thomson, *H.egregius* (Kokujev, 1909), *H.savioi* Uchida, 1940, *H.sugiharai* Uchida, 1936, and *H.tamahonis* (Uchida, 1931), have been known from China.

In the last two years the first two authors have been exploring the mountains in Qingdao (Laoshan Natural Reserve), Shandong Province, situated along the Yellow Sea in the northern border of the Oriental part of China, and have collected large numbers of ichneumonids. In this article, one new species, collected in this area is described.

## Materials and methods

Specimens were collected with interception traps (IT) proposed by Li et al. (2012) in Laoshan Natural Reserve, Qingdao, Shandong Province, P.R. China.

The type specimen of *Hoplocryptussavioi* Uchida, 1940, deposited in the Institute of Zoology, Chinese Academy of Sciences, Beijing, P.R. China, was examined. The photos of the types of *Aritranisohgushii* Momoi, 1963, *A.pini* Momoi, 1973, *Caenocryptusalboanalis* Uchida, 1952, *C.tamahonis* Uchida, 1931, *Hoplocryptusnigripeschinensis* Uchida, 1952, *H.sugiharai* Uchida, 1936 and *H.sumiyona* Uchida, 1956 (deposited in the Hokkaido University Museum and Museum of Nature and Human Activities, Sanda, Hyogo, Japan) taken by Dr. Kyohei Watanabe (Kanagawa Prefectural Museum of Natural History, Odawara, Japan: KPMNH), were checked and compared to the new species by the corresponding author.

Images were taken using a Leica M205A Stereomicroscope with LAS Montage MultiFocus. Morphological terminology is mostly based on Gauld (1991).

Type specimens are deposited in the Insect Museum, General Station of Forest and Grassland Pest Management (**GSFGPM**), National Forestry and Grassland Administration, People’s Republic of China.

### Hoplocryptus

Taxon classificationAnimaliaHymenopteraIchneumonidae

Thomson, 1873

b59a0f93-d6bd-40fc-95c8-7764100666e4

Hoplocryptus Thomson, 1873: 508.

#### Type-species.

*Hoplocryptusbinotatulus* Thomson, 1873 (= *murarius* Börner, 1782).

#### Diagnosis.

Ventral margin of clypeus with a more or less distinct tooth or tubercle (rarely somewhat paired). Mesoscutum with distinct punctation on a polished or subpolished background. Fore wing with sides of areolet subparallel or moderately narrowed anteriorly. Often both transverse carinae of propodeum entirely developed. Dorsolateral carina of first metasomal tergite usually distinct basal of spiracle (best seen in dorsal or dorsolateral view), its postpetiole rather weakly convex dorsally and not or only rather weakly wider than petiole. Second tergite with distinct and usually moderate sized punctures. Ovipositor compressed and its tip with rather regular and subvertical ridges on lower valve.

#### Remarks.

This genus morphologically resembles *Aritranis* in having sides of areolet parallel or moderately convergent anteriorly, 2m-cu straight or more or less sinuate, hind wing with M+Cu moderately to strongly arched, lateral longitudinal carina of propodeum absent, first metasomal segment without a lateral tooth basally and with its spiracle at or not very far behind its mid-length; but it can be distinguished from the latter by its dorsolateral carina of first metasomal tergite usually distinct basad of spiracle (best seen in dorsal or dorsolateral view), its postpetiole rather weakly convex dorsally, ventral margin of clypeus with a more or less distinct tooth (rarely paired teeth). Hosts are aculeate Hymenoptera. *Aritranis*: dorsolateral carina of first gastral tergite absent (or more rarely indistinct) basal of spiracle, postpetiole rather distinctly convex dorsally, ventral margin of clypeus without a tooth, except in the *Aritranisnigripes* group. Hosts are Lepidoptera and Coleoptera as far as known.

##### Key to the species of *Hoplocryptus* known from the Oriental and Eastern Palaearctic Regions (Female only)

This key does not include *H.egregius* (Kokujev, 1909) as its female is unknown.

**Table:** 

1	Clypeus with broad, blunt apical median tooth which has often a slight depression medially. Distance from vein 2rs-m to 2m-cu shorter than distance from 2m-cu to 3rs-m. Scutellum and median portion of hind tarsus often white. Tergites 2 and 3 red	***H.confector* (Gravenhorst)**
–	Clypeus with a narrow and pointed tooth, more rarely the tooth is somewhat blunt, tooth never with a depression medially. Differ often in other characters	**2**
2	Mesopleuron, mesosternum, propodeum and first tergite black, at most with white flecks	**3**
–	At least parts of propodeum, mesopleuron, mesosternum, or first tergite red or reddish yellow	**13**
3	Areolet convergent anteriorly. Ventral tooth of mandible as long as dorsal tooth. Tergites with dense large punctures. Mesosoma entirely black (sometimes red). Tergites 2–3(4) yellowish red	***H.heliophilus* (Tschek)**
–	Not entirely as above; if areolet convergent forward, then ventral tooth of mandible distinctly longer than dorsal tooth. Tergites with weak, fine punctures. Mesosoma with yellowish white spots. Tergites with distinct white spots	**4**
4	Lower tooth of mandible distinctly longer than upper tooth. Head posteriorly to eyes as seen from above strongly narrowed. Areolet convergent forward. Ovipositor tip long and comparatively low, about 4 times as long as high	***H.murarius* (Börner)**
–	Lower tooth of mandible usually as long as upper tooth. Head posteriorly to eyes as seen from above evenly narrowed. Areolet with vein 3rs-m approximately parallel to 2rs-m (except *H.alboanalis*). Ovipositor tip relatively short	**5**
5	Apical portion of dorsal valve of ovipositor (Fig. 10) with 6 small tubercles. Transverse carinae of propodeum (Fig. 7) complete and almost transversely straight. Lateral sides of face (Fig. 2) white. Posterior margins of all tergites (Fig. 8) distinctly white. Ventral profiles of hind coxae red brown, dorsal profile black	***H.qingdaoensis* Sheng, Wang & Schwarz, sp. nov.**
–	Apical portion of dorsal valve of ovipositor without tubercles (except *H.ohgushii*), rarely with indistinct one or two swellings. Transverse carinae of propodeum weak, posterior carina vestigial, or median portion of posterior transverse carina strongly bended forwards. Face entirely black. Tergites almost entirely black. Hind coxae unicolor	**6**
6	Metasomal tergites 2, 3, and hind leg black (hind femur of *H.quadriguttatus* red)	**7**
–	Tergites 2, 3 and hind femur reddish brown	**12**
7	Apical portion of scutellum white. Hind femur and tibia red	***H.quadriguttatus* (Gravenhorst)**
–	Scutellum and hind femur and tibia black (basal portion of *H.sugiharai* white)	**8**
8	Gena, mesosoma and metasomal tergites entirely covered with very dense and relatively large punctures. Hind wing vein 1-cu shorter than cu-a	***H.savioi* Uchida**
–	Gena, mesosoma and metasomal tergites with fine, relatively sparse punctures, at least tergites 4 to 6 with indistinct, fine punctures. Hind wing vein 1-cu at least as long as cu-a	**9**
9	Area basalis large, triangular. Distance between anterior transverse carina and posterior end of propodeum 3 times as long as distance from anterior transverse carina to anterior margin of area basalis. Apical portions of scutellum and first tergite and posterolateral portion of second tergite with white spots	***H.ohgushii* (Momoi)**
–	Area basalis relatively small, trapezoidal. Length between anterior transverse carina and posterior end of propodeum at least 4 times as long as distance from anterior transverse carina to anterior margin of area basalis. Scutellum, first and second tergites without white spots	**10**
10	Posterior transverse carina of propodeum complete, strongly arched forward medially. Area basalis distinctly longer than its width. Fore wing with vein 1cu-a opposite 1/M. Areolet convergent forward. Hind wing vein 1-cu 2.2 times as long as cu-a	***H.alboanalis* (Uchida)**
–	Posterior transverse carina of propodeum weak, slightly arched forward medially. Area basalis about as long as its width. Fore wing with vein 1cu-a basad of 1/M. Areolet with vein 3rs-m parallel to 2rs-m. Hind wing vein 1-cu at most 1.5 times as long as cu-a	**11**
11	Clypeus with dense punctures. Area basalis distinctly trapezoidal, strongly convergent backwardly. Tegula and hind tibia entirely black	***H.scorteus* (Momoi)**
–	Clypeus smooth, almost without punctures. Area basalis almost quadrate, nearly not convergent backwardly. Tegula and basal portion of hind tibia white	***H.sugiharai* Uchida**
12	Ovipositor sheath longer than hind tibia. Basal portion of clypeus with dense fine punctures. Tergites 2 and 3 with dense and large punctures. Hind coxa black	***H.femoralis* (Gravenhorst)**
–	Ovipositor sheath distinctly shorter than hind tibia. Clypeus shiny, basal portion with relative sparse fine punctures. Tergites with finely rugate and punctures. Hind coxa sometimes red	***H.coxator* (Tschek)**
13	Mesosoma 2.0–2.1 times as long as its maximum height. First tergite strongly arched medially. Hind coxa entirely brownish red. Posterior portions of tergites 2 and 3 with wide transverse white bands	***H.tamahonis* (Uchida)**
–	Mesosoma at most 1.9 times as long as its maximum height. First tergite arched distinctly beyond its middle. Hind coxa mainly black, at least apical portion more or less darkish. Tergites 2 and 3 without white bands	**14**
14	Antenna without white spot. Mesoscutum and mesopleuron usually orange. Hind coxa mainly black. Tergites 2 and 3 of metasoma almost entirely black	***H.bellosus* (Curtis)**
–	Antenna with white spot. Mesoscutum and at least anterior portion of mesopleuron black. Hind coxa mainly brown to reddish brown. Tergites 2 and 3 of metasoma orange to reddish brown	**15**
15	Mesoscutum with distinct punctures. Apical 0.7 of fore tibia strongly swollen. Posterior portion of mesopleuron brown	***H.pini* Momoi**
–	Mesoscutum extensively granulated. Fore tibia evenly, slightly swollen apically. Mesopleuron entirely black	***H.bohemani* (Holmgren)**

### Hoplocryptus qingdaoensis

Taxon classificationAnimaliaHymenopteraIchneumonidae

Sheng, Wang & Schwarz sp. nov.

15a28e40-6427-43da-9f6f-3ec440a7b1b1

http://zoobank.org/3B46329D-2E2A-4ADB-B631-496DA3B69DCA

Figures 1–10

#### Etymology.

The specific name is derived from the type locality.

#### Material examined.

Holotype. Female, CHINA: Laoshan, Qingdao, Shandong Province, 12 June 2017, IT. Paratype. 1 female, same data as holotype except 26 June 2017.

#### Diagnosis.

Propodeum rather long. Metasomal tergites 3–6 with even dense and unclear punctures. Second metasomal tergite (Figs 8, 9) slightly longer than its apical width. Ovipositor sheath 0.7 × as long as hind tibia. Apical portion of dorsal valve of ovipositor (Fig. 10) with 6 small tubercles. Face (Fig. 2) with large lateral white spots. All tergites (Fig. 8) with posterior white bands.

**Figures 1–10.**
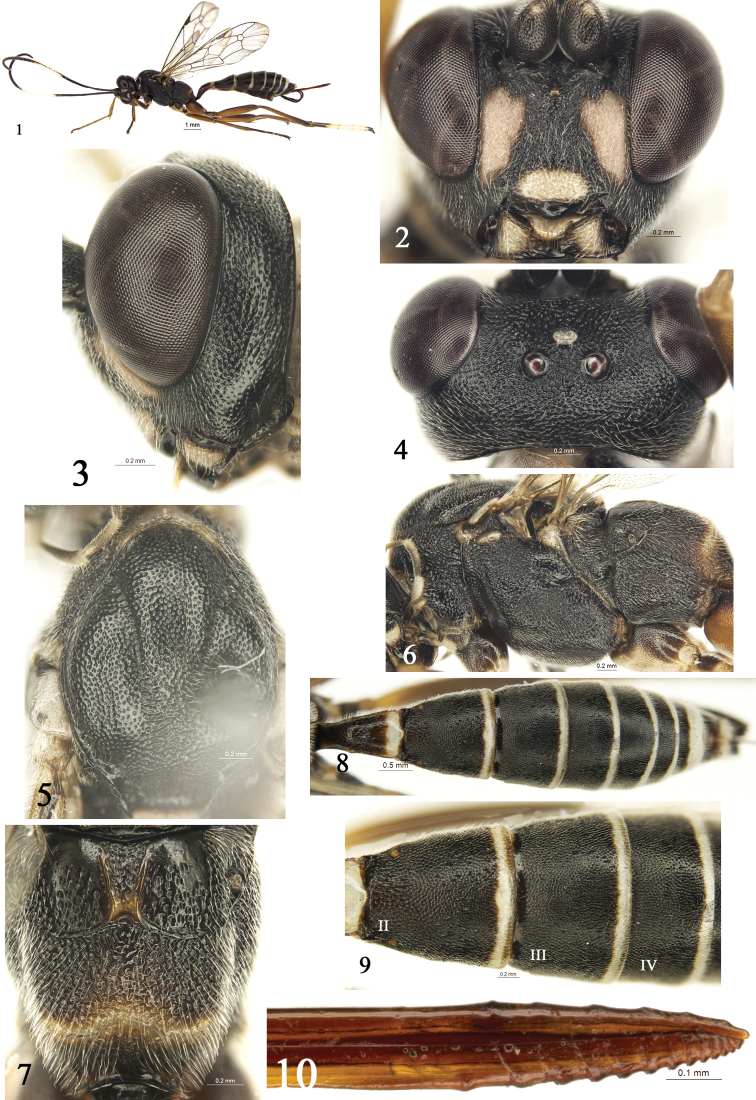
*Hoplocryptusqingdaoensis* Sheng, Wang & Schwarz, sp. nov. Holotype, female **1** habitus, lateral view **2** head, anterior view **3** head, lateral view **4** head, dorsal view **5** mesoscutum **6** mesosoma, lateral view **7** propodeum **8** metasoma, dorsal view **9** second to fourth tergites **10** apical portion of ovipositor, lateral view.

#### Description.

Female. Body length 11.0 to 11.9 mm. Fore wing length 7.0 to 7.4 mm. Ovipositor sheath length 2.3 to 2.4 mm.

#### Head.

Inner margins of eyes slightly convergent ventrally. Face (Fig. 2) medially slightly convex, 1.4 × as wide as long, with dense, indistinct punctures and opalescent hairs; lateral portion shagreened; upper margin with a small median tubercle. Clypeus 1.9 × as wide as long, slightly evenly convex; dorsal portion with dense punctures, ventrally smooth, ventral margin almost truncate, with a strong blunt tubercle medially (Fig. 2). Labrum almost semicircular, ventral margin with long brown hairs. Mandible with dense punctures and yellowish white hairs, upper tooth approximately as long as lower tooth. Malar area finely shagreened. Malar space 0.7 × as long as basal width of mandible. Gena with distinct punctures (Fig. 3). Vertex (Fig. 4) with dense distinct punctures. Postocellar line 0.8 × as long as ocular-ocellar line. Frons with a medio-longitudinal carina. Head behind the eyes in dorsal view weakly narrowed. Antenna with 32 flagellomeres. Ratios of lengths from first to fifth flagellomeres: 1.9:1.7:1.6:1.2:1.0. First flagellomere 8.0 × as long as wide. Occipital carina complete, reaching hypostomal carina distinctly above base of mandible. Hypostomal carina distinctly elevated.

#### Mesosoma.

Anterior portion of pronotum with dense yellowish white hairs, lateral concavity (Fig. 6) wide and shallow, subdorsal posterior portion with oblique rugae, upper and lower portions with dense indistinct punctures. Epomia distinct. Mesoscutum (Fig. 5) shiny, with dense punctures, postero-median portion with short transverse rugae. Notaulus evident on anterior half of mesoscutum. Scutoscutellar groove wide, with weak indistinct longitudinal rugae. Scutellum slightly convex, with sparse fine punctures. Postscutellum transversely slightly convex, shiny. Mesopleuron (Fig. 6) rugose, almost flat, with dense indistinct punctures; upper anterior portion beneath subalar prominence with short indistinct transverse rugae. Epicnemial carina weak, 0.8 × as long as mesopleuron. Speculum almost smooth, with sparse fine punctures. Sternaulus 0.4 × as long as length of mesopleuron. Metapleuron (Fig. 6) evidently rugose, with dense indistinct punctures and short hairs. Hind femur 5.4 × as long as maximum width. Ratio of length of one to fifth hind tarsomeres 6.3:2.9:1.8:1.0:1.8. Wings slightly grey, hyaline. Fore wing with vein cu-a basad of Rs & M by 0.3 times length of cu-a. Areolet receiving vein 2m-cu at its middle (Fig. 1). Vein 3rs-m approximately parallel to 2rs-m. Hind wing cu-a intercepted above middle. Propodeum rather long. Anterior and posterior transverse carinae of propodeum (Fig. 7) complete, median portions weakly bent forward. Area basalis shiny, lateral carinae distinct, strongly convergent caudally. Area externa shiny, anterior portion smooth, posterior portion with distinct punctures. Rest portions of propodeum densely irregularly reticulate, with dense short grey-white hairs. Propodeal spiracle small, almost circular.

#### Metasoma

(Figs 8, 9). First tergite 2.2 × as long as apical width; petiole and anterior portion of postpetiole almost shiny, with fine punctures; apical portion of postpetiole smooth, shiny, posteromedian portion distinctly convex. Median dorsal carina almost reaching to spiracle; dorsolateral and ventrolateral carinae complete. Tergites 2 to 6 with even dense distinct punctures. Second tergite (Figs 8, 9) slightly longer than its apical width. Third tergite 0.6 × as long as apical width. Ovipositor sheath 0.7 × as long as hind tibia. Ovipositor (Fig. 10) compressed; apical portion of dorsal valve with 6 small tubercles; apical portion of ventral valve with 13 edges.

#### Coloration

(Fig. 1). Black, except for the following. Large lateral spots of face, main portion of clypeus, labrum, median portion of mandible, median portions of maxillary and labial palpi, ventral profiles of flagellomeres (5)6 to 10(11), dorsoanterior portion of pronotum, scutellum, postscutellum, tegulae, subalar prominence, apex of fore coxa, ventral profile of mid coxa, apical half of hind tarsomere 1, hind tarsomeres 2–4, basal half of hind tarsomere 5, apical bands of all tergites white. Apical portions of fore femur, fore tibia and tarsus, ventral profiles of mid and hind femora and tibiae, ventral profiles of hind coxae red brown. Basal portion of fore femur, dorsal profiles of mid and hind femora, tibiae, hind coxa, apical portion of hind tibia brownish black. Lateral profile of first tergite slightly brown. Pterostigma and wing veins blackish brown.

#### Remarks.

This new species is similar to *Hoplocryptusalboanalis* (Uchida, 1952) by the characters: Clypeus with a strong smooth blunt tubercle medially; notaulus strong on anterior half of mesoscutum. anterior and posterior transverse carinae of propodeum complete; ventral valve of ovipositor with strong edges; mesosoma almost entirely black; median portion of flagellum, at least ventral profiles, white; and can be distinguished from the latter by the following combination of characters: Fore wing vein cu-a basad of Rs & M by about 0.3 times length of cu-a. Posterior transverse carina of propodeum (Fig. 7) evenly weakly bent forward. Face with large lateral white spots. Clypeus largely white. Ventral profile of hind coxa red brown, dorsal brownish black. Posterior portions of tergites with relative wide transverse white bands. *Hoplocryptusalboanalis*: Fore wing vein cu-a opposite Rs & M. Posterior transverse carina of propodeum almost sharply bent forward. Face, clypeus and hind coxa entirely black. Tergites entirely black.

## Supplementary Material

XML Treatment for Hoplocryptus

XML Treatment for Hoplocryptus qingdaoensis
